# Phlegmasia Dolens

**Published:** 1842-04

**Authors:** John Hardin

**Affiliations:** Horse-Well, Kentucky


### Art. II.—*Phlegmasia Dolens.* By John Hardin, M. D., of Horse-Well, Kentucky.

It is not my purpose at present to inquire whether phlegmasia dolens is an inflammation of the veins or absorbents, but to suggest a method of treatment which has been pursued with great satisfaction by myself, and also by my friend Dr. Jett, of Munfordville, a very accurate clinical observer, who has recently had an opportunity to test its efficacy.

In 1837, I was requested to visit Mrs. C., who had some days previously given birth to her third child, and had been for several hours the subject of swelled leg. The pain and swelling commenced at the ankle, and had already reached the groin. I immediately applied a strong cotton roller, comfortably tight, to the whole limb, commencing at the toes and terminating with a turn around the hips, to secure it *in situ*, and directed bathing with warm water to allay pain. The lady stated that she had bathed the limb, before my arrival, with warm whiskey, in which red pepper was steeped to saturation, and had found very considerable mitigation of pain therefrom; whereupon I directed the substitution of this mixture for the warm water. It gives me pleasure to say that the application always gave immediate relief. The experiment was frequently tried, without the patient's knowledge, of wetting the roller with warm spirit alone, but it did not allay the pain; and this was tried so frequently, and with the result so unvarying, that I was satisfied it could not be imaginary. In the constitutional treatment of this case, small portions of ipecacuanha were given at intervals of four hours, which operated as a cholagogue cathartic. No other medicine was used. Mercurials would have been administered in the beginning, but from the scorbutic appearance of the gums, the unpleasant local effects of the medicine were apprehended; and after the operations procured by the ipecacuanha were observed, nothing else was deemed necessary. In ten days the leg was well, there being only a slight, painless swelling remaining. At this time the disease attacked the other limb, and, under exactly similar treatment, was subdued in thirteen days.

Since that time, three other cases have been treated in the same manner (I mean the local treatment), and have terminated favorably, one in ten, and the two others in twelve days. In one of them, several doses of calomel and rhubarb were administered. Of course, in this, as in all other diseases, the internal remedies must be adapted to the constitutional disturbances present. In some of the cases the pain and swelling commenced in the groin, and progressed downwards to the foot.

In a disease attended with pain so excruciating as that under consideration, and one which renders the patient so utterly helpless, the advantage of the above method of treatment is incalculable, whether it be considered in reference to its power of allaying pain or of abridging the duration of the malady. The pain is absolutely under the immediate control of the remedy. In all the cases, about one gallon of the lotion was used in the first twenty-four hours, after which a quantity varying from a pint to a quart was found to be sufficient.

Believing that facts are more important as well as more acceptable to the profession, than speculations however ingenious, I submit these, unburthened with theoretical reflections.

Feb. 12th, 1842.

